# Communication between N terminus and loop2 tunes Orai activation

**DOI:** 10.1074/jbc.M117.812693

**Published:** 2017-12-13

**Authors:** Marc Fahrner, Saurabh K. Pandey, Martin Muik, Lukas Traxler, Carmen Butorac, Michael Stadlbauer, Vasilina Zayats, Adéla Krizova, Peter Plenk, Irene Frischauf, Rainer Schindl, Hermann J. Gruber, Peter Hinterdorfer, Rüdiger Ettrich, Christoph Romanin, Isabella Derler

**Affiliations:** From the ‡ Institute of Biophysics, Johannes Kepler University of Linz, Gruberstrasse 40, 4020 Linz, Austria, and; the § Center for Nanobiology and Structural Biology, Institute of Microbiology, Academy of Sciences of the Czech Republic, 373 33 Nove Hrady, Czech Republic

**Keywords:** atomic force microscopy (AFM), calcium release-activated calcium channel protein 1 (ORAI1), electrophysiology, signal transduction, stromal interaction molecule 1 (STIM1)

## Abstract

Ca^2+^ release-activated Ca^2+^ (CRAC) channels constitute the major Ca^2+^ entry pathway into the cell. They are fully reconstituted via intermembrane coupling of the Ca^2+^-selective Orai channel and the Ca^2+^-sensing protein STIM1. In addition to the Orai C terminus, the main coupling site for STIM1, the Orai N terminus is indispensable for Orai channel gating. Although the extended transmembrane Orai N-terminal region (Orai1 amino acids 73–91; Orai3 amino acids 48–65) is fully conserved in the Orai1 and Orai3 isoforms, Orai3 tolerates larger N-terminal truncations than Orai1 in retaining store-operated activation. In an attempt to uncover the reason for these isoform-specific structural requirements, we analyzed a series of Orai mutants and chimeras. We discovered that it was not the N termini, but the loop2 regions connecting TM2 and TM3 of Orai1 and Orai3 that featured distinct properties, which explained the different, isoform-specific behavior of Orai N-truncation mutants. Atomic force microscopy studies and MD simulations suggested that the remaining N-terminal portion in the non-functional Orai1 N-truncation mutants formed new, inhibitory interactions with the Orai1-loop2 regions, but not with Orai3-loop2. Such a loop2 swap restored activation of the N-truncation Orai1 mutants. To mimic interactions between the N terminus and loop2 in full-length Orai1 channels, we induced close proximity of the N terminus and loop2 via cysteine cross-linking, which actually caused significant inhibition of STIM1-mediated Orai currents. In aggregate, maintenance of Orai activation required not only the conserved N-terminal region but also permissive communication of the Orai N terminus and loop2 in an isoform-specific manner.

## Introduction

The essential molecular components of Ca^2+^ release-activated Ca^2+^ (CRAC)^4^ signaling are represented by the Ca^2+^ sensor protein STIM1, which is anchored in the endoplasmic reticulum membrane (1, 2) and the pore-forming subunit Orai in the plasma membrane (3–5). CRAC channel gating involves STIM1 multimerization, a subsequent redistribution into puncta (1, 2, 6), and direct binding of the STIM1 C terminus to both cytosolic termini of the Orai channel (7–9). The Orai C terminus is the main interaction partner for the STIM1 C terminus, whereas direct coupling of STIM1 to the N terminus is currently controversial (10–14).

Orai proteins (Orai1–3) represent highly Ca^2+^-selective channels (7, 15, 16). All three homologues display a high inward rectification and a low unitary conductance, whereas major differences in terms of fast Ca^2+^-dependent inactivation, their response to 2-aminoethoxydiphenyl borate, and their redox sensitivity have also been reported (17–26).

The crystal structure of Orai from *Drosophila melanogaster* (dOrai) (27) displays a hexameric Orai stoichiometry. Transmembrane domains 1 (TM1) line the pore (28) (29), form the inner of three concentric rings, and extend into the cytosol by four helical turns (27), the so-called conserved extended transmembrane Orai N terminus (ETON) region (13). The second ring consists of transmembrane regions 2 and 3, and the outer ring is formed by TM4 (27). TM2 has been reported to traverse the lipid bilayer just beyond its thickness, and TM3 protrudes into the cytosol by two helical turns as well (27). The flexible linker connecting TM2 and TM3 (*i.e.* loop2) has not been resolved in the crystal structure of dOrai.

STIM1 coupling to the Orai channel opens the pore via a mechanism involving rotation of the pore helix (30). However, just how this conformational change to the open state of the pore occurs has so far remained elusive. Several constitutively active mutants containing substitutions not only in TM1 (Gly^98^, Phe^99^, and Val^102^ (12, 13, 30–32)), but also in other TM regions (Leu^138^ (33) in TM2, Trp^176^ in TM3 (34), P245L in TM4 (35), and Leu^261^-Val^262^-His^264^-Lys^265^ in the hinge connecting TM4 and the C terminus (14)), suggest that Orai channels capture the open state via a global rearrangement of all TM helices.

Ca^2+^ ions enter the Orai pore upon their attraction via three aspartates in the first extracellular loop region that functions as a Ca^2+^-accumulating region (21, 29), termed CAR (36). They pass the narrow opening of 6 Å at Glu^106^ forming the Ca^2+^ selectivity filter followed by a hydrophobic segment including Val^102^, Phe^99^, and Gly^98^ (27, 37). Their substitution results in constitutively open and non-selective Ca^2+^ currents in the absence of STIM1, which become selective in the presence of STIM1, comparable with wildtype Orai1 (31), and thus the reversal potential can be used as a readout parameter for STIM1 binding (12, 13). Another narrow part of the pore is formed by the basic segment at the TM1-N terminus interface. Substitution of Arg^91^ for tryptophan leads to a block of the pore by the bulky, hydrophobic side chains (37).

The conserved, helical ETON region (aa 73–90 of Orai1) (13) has already been reported to be indispensable for Orai gating, based on several truncation and point mutants (10, 12, 13, 38, 39) and is involved in regulation via cholesterol (40). Intriguingly, Orai3 requires approximately one and a half helix turn less of the ETON region compared with Orai1 for store-operated activation, although their ETON regions are fully conserved (13, 38), suggesting distinct molecular determinants in the activation of Orai1 and Orai3 channels.

The aim of this study was to clarify the reason for the distinct isoform-specific structural requirements between N-truncated Orai1 and Orai3 channels in maintaining function. We discovered that non-functional Orai1 N-truncation mutants regained function upon swapping the loop2 segment, connecting TM2 and TM3, with that of Orai3. Mechanistically, we uncovered a distinct behavior between Orai1-loop2 and Orai3-loop2, with the former leading to inhibitory interactions with the truncated N terminus and to non-functional Orai1 channels. We suppose that maintenance of Orai channel function requires permissive communication between the N terminus and loop2, probably governed in an isoform-specific manner.

## Results

### Non-functional Orai1 N-terminal deletion/point mutants regain function by swapping loop2 with that of Orai3

**Figure 1.**
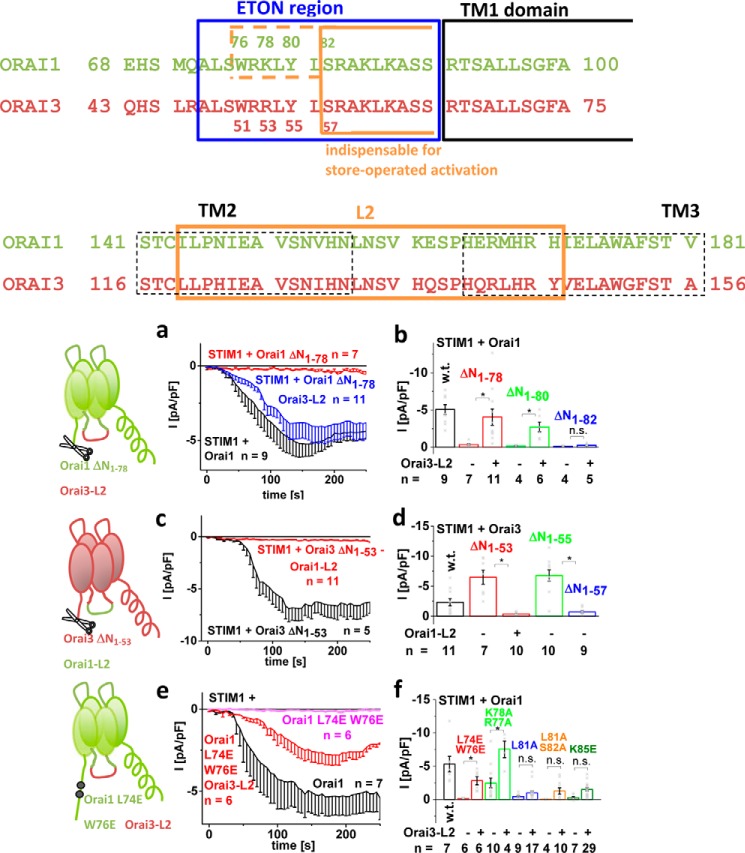
**Non-functional Orai1 N-terminal deletion/point mutants regain function by swapping loop2 with that of Orai3.** Shown is the sequence alignment of Orai1 and Orai3 N terminus and loop2. *a*, time course of whole-cell inward currents at −74 mV activated upon passive store depletion of HEK 293 cells co-expressing STIM1 + Orai1 ΔN_1–78_ Orai3-loop2 in comparison with Orai1 and Orai1 ΔN_1–78_. *b*, block diagram showing STIM1-mediated maximal currents at *t* = 180 s upon whole-cell break-in of wildtype Orai1 compared with Orai1 ΔN_1–78_, Orai1 ΔN_1–78_ Orai3-loop2, Orai1 ΔN_1–80_, Orai1 ΔN_1–80_ Orai3-loop2, Orai1 ΔN_1–82_, and Orai1 ΔN_1–82_ Orai3-loop2. *c*, time course of whole-cell inward currents at −74 mV activated upon passive store depletion of HEK 293 cells co-expressing STIM1 + Orai3 ΔN_1–53_ Orai1-loop2 in comparison with Orai3 ΔN_1–53_. *d*, block diagram showing STIM1-mediated maximal currents at *t* = 180 s upon whole cell break-in of wildtype Orai3 compared with Orai3 ΔN_1–53_, Orai3 ΔN_1–53_ Orai1-loop2, Orai3 ΔN_1–55_, and Orai3 ΔN_1–57_ Orai1-loop2. *e*, time course of whole-cell inward currents at −74 mV activated upon passive store depletion of HEK 293 cells co-expressing STIM1 + Orai1 L74E/W76E Orai3-loop2 in comparison with Orai1 and Orai1 L74E/W76E. *f*, block diagram showing STIM1-mediated maximal currents at *t* = 180 s upon whole-cell break-in of wildtype Orai1 compared with Orai1 L74E/W76E, Orai1 L74E/W76E Orai3-loop2, Orai1 K77A/R78A, Orai1 K77A/R78A Orai3-loop2, Orai1 L81A, Orai1 L81A Orai3-loop2, Orai1 L81A/S82A, Orai1 L81A/S82A Orai3-loop2, Orai1 K85E, and Orai1 K85E Orai3-loop2. *Error bars*, S.E.; *, *p* < 0.05; *n.s.*, not significant.

Activation of Orai channels requires, in addition to the C terminus, the Orai N terminus (12, 13). The minimal portion of the conserved ETON region, indispensable for retaining STIM1-dependent activation, is distinct between Orai1 and Orai3 (alignment in Fig. 1 (*top*)) (13, 38). Here, we initially focused on the distinct, isoform-specific structural requirements between N-truncated Orai1 and Orai3 channels in maintaining STIM1-mediated function. Intriguingly, whereas the Orai1 N-terminal deletion mutants Orai1 ΔN_1–76/78_ lost function (Fig. 1, *a* and *b*) (13), the analogue Orai3 N-truncation mutants (Orai3 ΔN_1–51/53_) maintained STIM1-dependent activation (38) (Fig. 1, *c* and *d*). In other words, Orai3 required a 5-residue shorter portion of the ETON region than Orai1, despite the fact that this region is fully conserved (13, 38) (Fig. 1 (*b* and *d*) and Fig. S1), suggesting isoform-specific structural requirements. Orai3 only lost STIM1-dependent activation upon deletion of the first 57 N-terminal residues (38), which corresponded to the first 82 N-terminal residues of Orai1 (Fig. 1 (*b* and *d*) and Fig. S1). We aimed at identifying the molecular mechanism behind the loss of Orai1 ΔN_1–78_ function and the reason for these distinct structural requirements in the Orai1 and Orai3 activation by employing a chimeric approach.

Generation of N-truncated Orai1-Orai3 chimeras revealed that among the non-conserved regions of Orai proteins that were exchanged in the non-functional Orai1 N-truncation mutants by the respective ones of Orai3 (Orai3-loop2-aa 116–156, Orai3-loop2-aa 119–147, Orai3-loop2-aa 136–141, Orai3 TM3, Orai3-loop3, Orai3 TM4, and Orai3 C terminus; Fig. S2), the Orai3 C terminus was sufficient to restore function of Orai1 ΔN_1–76_ but failed with the shorter Orai1 N-truncation mutant, Orai1 ΔN_1–78_ (Fig. S2, *a* and *b*). Chimeric Orai1 N-truncation mutants containing TM3, loop3, and/or TM4 exchanged by that of Orai3 likewise left Orai1 ΔN_1–78_ inactive (Fig. S2*c*). However, when swapping the Orai1-loop2 with that of Orai3 (Orai3-loop2-aa 119–147 or Orai3-loop2-aa 116–156; alignment in Fig. 1 (*bottom*)), including the helical extensions of TM2 and TM3 with a flexible portion in between, STIM1-dependent activation of Orai1 ΔN_1–76/78/80_ (Orai1 ΔN_1–78/80_ Orai3-loop2; Fig. 1 (*a* and *b*) and Fig. S2 (*a* and *c*)) was restored, but not that of Orai1 ΔN_1–82_ (Orai1 ΔN_1–82_ Orai3-loop2) (Fig. 1, *a* and *b*). This result was fully in line with the maintained or lost function of the analogue Orai3 deletion mutants Orai3 ΔN_1–53/55_ or Orai3 ΔN_1–57_ (Fig. 1, *c* and *d*), respectively. These functional chimeras behaved with respect to the typical CRAC channel hallmarks comparably with analogue Orai3 N-truncation mutants, as shown for Orai1 ΔN_1–78_ Orai3-loop2 and Orai3 ΔN_1–53_, respectively (71). The analogue Orai3 N-truncation mutant (Orai3 ΔN_1–53_) conversely lost function upon the exchange of its loop2 with that of Orai1 (Orai1-loop2-aa 144–172 or Orai1-loop2-aa 141–181; alignment in Fig. 1 (*bottom*)) (Fig. 1*b* and Fig. S2*d*). The STIM1 C-terminal fragments (*i.e.* the Orai-activating STIM1 fragment (OASF) (aa 233–474) and OASF-L251S) correspondingly induced constitutive activation of Orai1 ΔN_1–78_ Orai3-loop2 and left Orai3 ΔN_1–53_ Orai1-loop2 inactive (Fig. S2, *e–g*). In support of this, impaired N-terminal point mutants up to Lys^78^ (Orai1 L74E/W76E, Orai1 R77A/K78A) displayed strong recovery in Orai1 channel function upon an exchange of loop2, whereas those downstream (Orai1 L81A, Orai1 L81A/S82A, and Orai1 K85E) regained only marginal activation (Fig. 1, *e* and *f*). Distortions via endogenous STIM1 were less likely, as STIM1-dependent activation of Orai1 ΔN_1–78_ could be similarly restored upon the swap of Orai3-loop2 in CRISP/Cas STIM1 knockout HEK cells (Fig. S2*h*).

In summary, these experiments clearly revealed that loop2 of Orai3 restored function of non-functional Orai1 N-truncation mutants and N-terminal point mutants, especially with substitutions in the first (aa 73–81) but not the second half of the conserved ETON region. Hence, isoform-specific properties of Orai1- and Orai3-loop2 in combination with the truncated or mutated N-terminal portion were probably responsible for isoform-specific activation. Furthermore, these results suggested that only the second half of the ETON region was indispensable for store-operated activation, in both Orai1 and Orai3 channels.

### Mutation of five non-conserved residues in loop2 of Orai1 is sufficient to convey STIM1-dependent activation onto non-functional Orai1 N-truncation mutants

**Figure 2.**
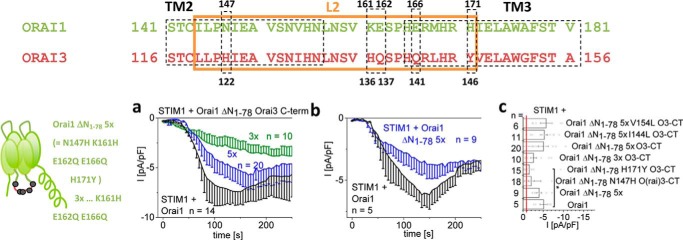
**Mutation of five non-conserved residues in loop2 of Orai1 is sufficient to convey STIM1-dependent activation onto non-functional Orai1 N-truncation mutants.** Shown is the sequence alignment of Orai1- and Orai3-loop2 highlighting distinct residues that were mutated in Orai1 ΔN_1–78_. *a*, time course of whole-cell inward currents at −74 mV activated by passive store depletion of HEK 293 cells co-expressing STIM1 + Orai1 ΔN_1–78_ N147H/K161H/E162Q/E166Q/H171Y in comparison with STIM1 + Orai1. *b*, time course of whole-cell inward currents at −74 mV activated by passive store depletion of HEK 293 cells co-expressing STIM1 and Orai1 ΔN_1–78_ N147H/K161H/E162Q/E166Q/H171Y Orai3-C-term or Orai1 ΔN_1–78_ K161H/E162Q/E166Q Orai3-C-term in comparison with STIM1 + Orai1. *c*, block diagram showing STIM1-mediated maximal currents at *t* = 180 s upon whole-cell break-in of wildtype Orai1 compared with Orai1 ΔN_1–78_ 5×, Orai1 ΔN_1–78_ N147H Orai3-C-term, Orai1 ΔN_1–78_ H171Y Orai3-C-term, Orai1 ΔN_1–78_ 3× Orai3-C-term, Orai1 ΔN_1–78_ 5× Orai3-C-term, Orai1 ΔN_1–78_ 5× I144L Orai3-C-term, and Orai1 ΔN_1–78_ 5× V154L Orai3-C-term. *Error bars*, S.E.

The loop2 regions of Orai1 (aa 144–172) and Orai3 (aa 119–147) are conserved up to 75% (alignment in Fig. 2 (*top*); Fig. S2), suggesting that only a few different amino acids are responsible for the dramatic functional differences of the Orai1 and Orai3 N-truncation mutants. We performed site-directed mutagenesis of residues in loop2 of Orai1 ΔN_1–78_ with properties strongly distinct from those at analogue positions in Orai3 (Fig. 2). Among single or multiple point mutations, mimicking loop2 residues in Orai3, only the mutation of a set of five residues (N147H/K161H/E162Q/E166Q/H171Y (5×)) restored significant, STIM1-dependent activation (Fig. 2, *a* and *c*), similar to Orai1 ΔN_1–78_ Orai3-loop2. Substitution of more than 5 distinct residues did not further enhance currents (Fig. 2, *a* and *c*). Initially, each of these loop2 point mutations was introduced in an Orai1 ΔN_1–78_ Orai3-C-term background. As Orai1 ΔN_1–78_ remained non-functional upon the mere swap of the Orai3-C-term, potential recovery of activity was most likely linked to the additionally introduced loop2 mutations. The 5-fold loop2 point mutation inserted in Orai1 ΔN_1–78_ (Orai1 ΔN_1–78_ N147H/K161H/E162Q/E166Q/H171Y) consistently yielded significant current activation almost comparable with wildtype Orai1 (Fig. 2, *a–c*) or Orai1 ΔN_1–78_ Orai3-loop2 (Fig. S2*c*), independent of the Orai3-C-term. This behavior was widely reproduced upon activation of these multiple point mutants by the STIM1 C-terminal fragment OASF-L251S (Fig. S2, *h* and *i*). In total, except for five non-conserved residues in loop2, not a single point mutation was sufficient to fully restore function of Orai1 Δ_N1–78_.

### Functional Orai1 ΔN_1-78_ Orai3-loop2 chimera displays recovered coupling to STIM1

**Figure 3.**
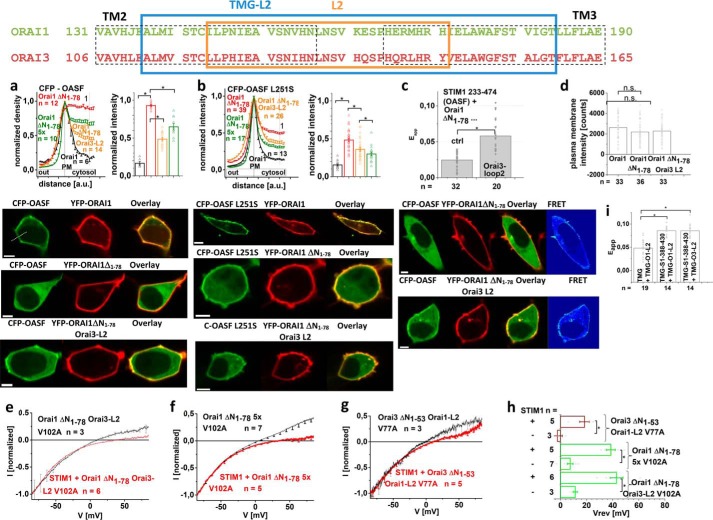
**Functional Orai1 ΔN_1–78_ Orai3-loop2 chimera displays recovered coupling to STIM1.** *Top*, sequence alignment of Orai1- and Orai3-loop2 depicting loop2 fragment used in the chimeras (*framed* in *orange*) and the one attached to TMG used for FIRE measurements (*framed* in *blue*). *a* and *b*, intensity plots representing the localization of STIM1 233–474 (*a*) and STIM1 233–474 L251S (*b*) across the cell when co-expressed with Orai1, Orai1 ΔN_1–78_, Orai1 ΔN_1–78_ Orai3-loop2, and Orai1 ΔN_1–78_ 5× together with bar graphs exhibiting normalized intensities at point 1. Image series depict CFP-OASF/-OASF L251S and YFP-Orai1, -Orai1 ΔN_1–78_, or -Orai1 ΔN_1–78_ Orai3-L2 and *overlay. c*, bar graph depicting FRET of STIM1 233–474 with Orai1 ΔN_1–78_ and Orai1 ΔN_1–78_ Orai3-loop2. Image series depict CFP-OASF and YFP-Orai1, -Orai1 ΔN_1–78_, or Orai1 ΔN_1–78_ Orai3-L2, *overlay*, and *pixelwise* calculated *N*_FRET_ index for a representative cell. *Yellow arrows*, plasma membrane localization. *d*, block diagram exhibiting the mean plasma membrane intensities of Orai1, Orai1 ΔN_1–78_, and Orai1 ΔN_1–78_ Orai3-loop2 in comparison. *e*, *I*/V relationship of normalized Orai1 ΔN_1–78_ Orai3-loop2 V102A with and without STIM1. *f*, *I*/V relationships of normalized Orai1 ΔN_1–78_ 5× V102A with and without STIM1. *g*, *I*/V relationships of normalized Orai3 ΔN_1–53_ Orai1-loop2 V77A with and without STIM1. *h*, block diagram displaying the reversal potentials of Orai1 ΔN_1–78_ Orai3-loop2 V102A, Orai1 ΔN_1–78_ 5× V102A, and Orai3 ΔN_1–53_ Orai1-loop2 V77A currents in the absence compared with the presence of STIM1. *i*, bar graph depicting FRET of fluorescently labeled CFP-TMG-STIM1 388–430 with YFP-TMG-Orai1-loop2 or YFP-TMG-Orai3-loop2 in comparison with CFP-TMG with YFP-TMG-Orai1-loop2. *Error bars*, S.E.; *, *p* < 0.05; *n.s.*, not significant.

Regained STIM1-dependent function of the Orai1 ΔN_1–78_-Orai3-loop2 chimera suggested that coupling to STIM1 was primarily restored. Co-localization studies indeed revealed restored coupling of the STIM1 C-terminal fragments OASF or OASF L251S to Orai1 ΔN_1–78_ Orai3-loop2 and also the Orai1 ΔN_1–78_ 5-fold loop2 point mutant (Orai1 ΔN_1–78_ 5×) in comparison with Orai1 ΔN_1–78_, although to a lower extent than for wildtype Orai1 (Fig. 3, *a* and *b*). In agreement, FRET measurements of STIM1-OASF with Orai1 ΔN_1–78_ Orai3-loop2 displayed increased coupling when compared with Orai1 ΔN_1–78_ (Fig. 3*c*). Fluorescence intensity measurements revealed comparable plasma membrane expression of Orai1, Orai1 ΔN_1–78_, and Orai1 ΔN_1–78_ Orai3-loop2 (Fig. 3*d*).

Further, we utilized a potential rightward shift of the reversal potential of the constitutively active Orai1 V102A mutant and the analogue Orai3 V77A as a readout parameter for STIM1 binding. Whereas proper STIM1 binding correlated with a reversal potential of >+40 mV, reduced STIM1 binding or its total loss left the reversal potential at more negative values (at about 10–20 mV) (13), which occurred in an analogue manner for Orai3 V77A (Fig. S3). In contrast to Orai1 ΔN_1–78_ V102A, which displayed non-selective currents with a reversal potential around ∼+10 mV also in the presence of STIM1 (13), Orai1 ΔN_1–78_ Orai3-loop2 V102A exhibited a rightward shift in the reversal potential to ∼+40 mV in the presence of STIM1 (Fig. 3, *e* and *h*). In line with this, Orai1 V102A with the 5-fold loop2 mutation resulted in constitutively active, non-selective currents in the absence of STIM1, whereas in the presence of STIM1, the reversal potential shifted to more positive values (∼+40 mV) (Fig. 3, *f* and *h*), suggesting recovered STIM1 coupling. Conversely, Orai3 ΔN_1–53_ Orai1-loop2 V77A displayed much less of an increase in the reversal potential when STIM1 was co-expressed (Fig. 3, *g* and *h*), in contrast to the highly Ca^2+^-selective currents of Orai3 ΔN_1–53_ V77A in the presence of STIM1 (Fig. S3), pointing to an inhibitory impact of Orai1-loop2 on the coupling with STIM1.

Enhanced STIM1 coupling to Orai1 ΔN_1–78_ upon the exchange with Orai3-loop2 suggested an impact of loop2 on the overall STIM1 coupling. It is of note that Park *et al.* (41) have not detected any coupling of the CRAC activation domain of STIM1 to Orai1-loop2 (aa 141–177), in line with our observations (data not shown). However, by employing the FRET-derived interaction in a restricted environment (FIRE) system (42), a larger Orai1-loop2 fragment with a few additional residues on both the N-terminal and the C-terminal side (aa 137–184) revealed significant coupling with a portion (TMG-STIM1-aa 388–430) of STIM1-OASF (aa 233–474). Robust FRET (Fig. 3*i*) was obtained for both Orai1-loop2 and Orai3-loop2 to a similar extent (Fig. 3*i*), suggesting that the distinct function of Orai1 and Orai3 N-terminal truncation mutants was less likely to be caused by isoform-specific loop2 coupling to STIM1.

Overall, restored STIM1 coupling with Orai1 ΔN_1–78_ Orai3-loop2 corresponded with the recovery of function, both of which were lost with Orai1 ΔN_1–78_. Similar coupling of a STIM1 fragment to Orai1-loop2 or Orai3-loop2, however, might point to an allosteric, indirect effect of Orai1-loop2, which affected Orai activation in a distinctly different, isoform-specific manner. In an attempt to investigate whether such an effect is confined to the Orai channel itself, we examined the effects of loop2 on Orai N-truncation mutants that function in a STIM1-independent manner.

### Lost constitutive activity of N-truncated Orai1 mutants is restored by Orai3-loop2

**Figure 4.**
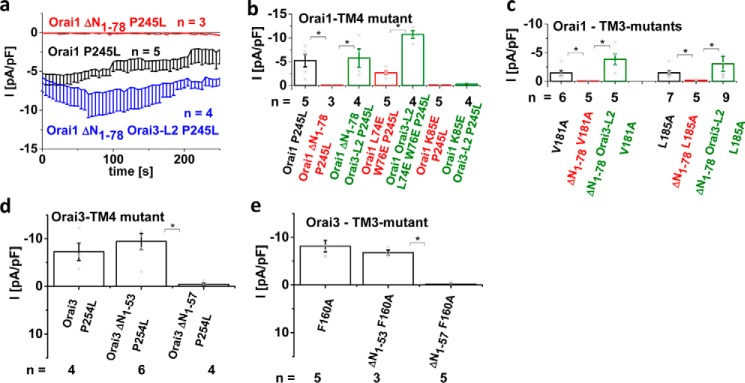
**Lost constitutive activity of N-truncated Orai1 mutants is restored by Orai3-loop2.** *a*, time course of whole-cell inward currents at −74 mV activated by passive store depletion of HEK 293 cells expressing Orai1 P245L compared with Orai1 ΔN_1–78_ P245L and Orai1 ΔN_1–78_ Orai3-loop2 P245L. *b*, bar graphs comparing constitutive currents at *t* = 0 s after whole-cell break-in of Orai1 P245L, Orai1 ΔN_1–78_ P245L, Orai1 ΔN_1–78_ Orai3-loop2 P245L, Orai1 L74E/E76E/P245L, Orai1 L74E/E76E Orai3-loop2 P245L, Orai1 K85E/P245L, and Orai1 K85E/P245L Orai3-loop2. *c*, bar graphs comparing constitutive currents at *t* = 0 s after whole-cell break-in of Orai1 V181A, Orai1 ΔN_1–78_ V181A, Orai1 ΔN_1–78_ Orai3-loop2 V181A, Orai1 L185A, Orai1 ΔN_1–78_ L185A, and Orai1 ΔN_1–78_ Orai3-loop2 L185A. *d*, bar graphs comparing constitutive currents at *t* = 0 s after whole-cell break-in of Orai3 F160A, Orai3 ΔN_1–53_ F160A, and Orai3 ΔN_1–57_ F160A. *e*, bar graphs comparing constitutive currents at *t* = 0 s after whole-cell break-in of Orai3 P254L, Orai3 ΔN_1–53_ P254L, and Orai3 ΔN_1–57_ P254L. *, *p* < 0.05. *Error bars*, S.E.

To investigate whether the effects of Orai1-loop2 and Orai3-loop2 also occurred solely at the level of the Orai proteins in the absence of STIM1, we utilized several constitutively active Orai mutants containing substitutions in TM3 and/or TM4. Initially, investigation of the constitutively active Orai1 mutant Orai1 P245L, which is associated with tubular myopathy (43), revealed loss of constitutive activity upon N-terminal truncation up to Lys^78^ (*i.e.* Orai1 ΔN_1–78_ P245L) (Fig. 4, *a* and *b*). Indeed, the swap of Orai1-loop2 with that of Orai3 recovered constitutive activation of this truncated point mutant (see also Ref. 71). Analogously, constitutively active Orai1 TM3 mutants (Orai1 V181A and Orai1 L185A) (Fig. 4*c*) lost function upon N-truncation up to Lys^78^, whereas currents were restored by the swap of Orai3-loop2. In line with this, analogue Orai3 N-truncation mutants (Orai3 ΔN_1–53_) remained active as exemplarily shown for Orai3 P254L (the analogue of Orai1 P245L) and Orai3 F160A (the analogue of Orai1 L185A) (Fig. 4, *d* and *e*; see also Ref. 71), whereas only Orai3 ΔN_1–57_ mutants lost function.

Introduction of the double point mutant L74E/W76E in Orai1 P245L did not completely abolish, but significantly reduced, constitutive currents, which were enhanced by the Orai3-loop2 in Orai1 (Orai1 L74E/W76E Orai3-loop2 P245L) (Fig. 4*b*). In contrast, the single point mutation K85E not only leaves Orai1 P245L but also Orai1 Orai3-loop2 P245L inactive in the absence of STIM1, highlighting Lys^85^ as essential for general Orai channel function also in support of Ref. 39 (see also Ref. 71).

In summary, constitutive Orai1 mutants lost function in the N-terminal truncated Orai1 ΔN_1–78_ form; however, their activity was restored upon the swap of Orai3-loop2. These results pointed to an inhibitory effect of Orai1-loop2 in the N-truncated mutants, which occurred independent of STIM1. Hence, we supposed that Orai1-loop2 and Orai3-loop2 induced distinct, isoform-specific Orai conformations, permissive or non-permissive, that accounted for the functional differences and additionally probably affected STIM1 binding in an indirect manner.

### In comparison with the N-terminal peptide Orai1-NT 70-91, the shorter Orai1-NT 79-91 shows stronger and more frequent interactions with Orai1-loop2

**Figure 5.**
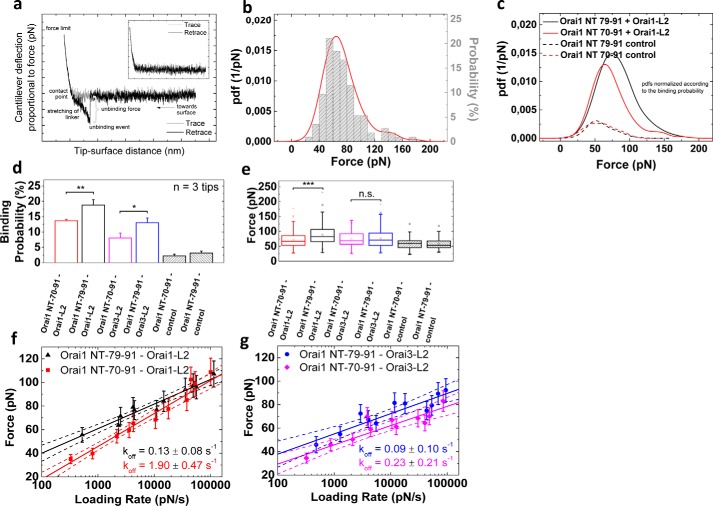
**AFM single-molecule force spectroscopy reveals significant higher interaction forces and lower dissociation rates between truncation mutant Orai1 ΔN_1–78_ and Orai1-L2.** *a*, exemplary force–distance curve and distribution of unbinding forces. In the beginning, the cantilever with the tip-tethered ligand is far away from the surface with immobilized receptors (see *pictogram*), and no force is exerted on the cantilever. By continuously approaching the tip to the surface (*trace*, *gray line*), the tip comes in contact with the surface (contact point) and is bent upward, resulting in an increase in force until a certain force value (force limit) is reached. If an interaction between the tip-tethered molecule and the surface-bound molecule occurred, the tip is bent downward during tip retraction (*retrace*, *black line*), and stretching of the PEG linker will cause a typical parabolic force profile. The force at which the complex dissociates and the cantilever jumps back to its resting position directly corresponds to the unbinding force of the respective interaction. Force–distance curves where no interaction occurs show very similar trace and retrace curves with no characteristic nonlinear stretching of the PEG linker (*inset*, no ligand is tethered to the tip). *b*, as an empirical estimate, the pdf (*red line*) of all unbinding forces within one data set is calculated and allows for determination of the most probable unbinding force with better resolution compared with a conventional histogram (*gray bars*). Shown is the pdf of the interaction between tip-tethered Orai1 L2 and surface-immobilized Orai1-NT 70–91. *c*, *overlay* of the force distributions of the interactions between tip-tethered Orai1 L2 and surface-immobilized Orai1-NT 79–91 (*black solid line*) or Orai1-NT 70–91 (*red solid line*, as in *b*). The pdfs that are shown as *dashed lines* represent the respective control experiment without Orai1 L2 on the tip. All pdfs were normalized to the highest binding probability (*i.e.* Orai1-NT 79–91; *black solid line*); the *area under* each pdf therefore represents the respective binding probability in relation to the one of Orai1-NT 79–91. *d*, binding probability between surface-immobilized Orai1 70–91 or Orai1 79–91 and tip-tethered Orai1-L2 or Orai3-L2, respectively. Control experiments were performed with unfunctionalized tips carrying only the cross-linker (*striped bars*). *e*, box plot of the prevailing interaction forces at a loading rate of ∼5000 pN/s. *f* and *g*, plot of interaction force *versus* loading rate for the interaction of Orai1 70–91 and Orai1 79–91 with Orai1-L2 (*f*) and Orai3-L2 (*g*). For the sake of clarity, error bars for the S.D. of loading rates are not shown but were considered for the fitting procedure. Together with the fit (*solid lines*), confidence bands (95% confidence interval) of the respective fits are illustrated by *dotted lines*. *, *p* < 0.05; **, *p* < 0.01; ***, *p* < 0.001.

The observed effects in constitutive mutants suggested that the N-terminal truncated Orai1 forms displayed an altered interplay of the N terminus and loop2 in contrast to the full-length or N-truncated Orai3-loop2 chimeric proteins. To examine whether the longer or the shorter N terminus (NT) might form altered interactions with loop 2 (L2) from either Orai1 or Orai3, possibly accounting for the inhibitory effects on Orai1 channel activation, we performed *in vitro* force measurements using atomic force microscopy (AFM). For this purpose, isolated Orai1-L2 or Orai3-L2 was covalently conjugated onto the apex of the AFM cantilever tip via a flexible PEG linker. Single molecular force measurements were performed by lowering the functionalized tip toward surface-bound Orai1-NT 70–91 or Orai1-NT 79–91, followed by subsequent retraction. In the case of an interaction, a pulling force was developed during retraction, causing a downward bending of the cantilever. At a certain critical force, the bond between the two fragments was ruptured (Fig. 5*a*), allowing for quantitative determination of the single molecular unbinding force (Fig. 5, *b* and *c*). The number of force–distance curves that showed unbinding events (*i.e.* binding probability) for both surface-immobilized Orai1-NT 70–91 and Orai1-NT 79–91 decreased significantly when performing the same experiments with tips only carrying the PEG linker without loop2 (*striped bars* in Fig. 5*d*), proving the specificity of the measured interactions. Our measurements indicate that the shorter Orai1-NT fragment leads to an increased probability for the interaction with loop2, showing statistically stronger evidence for the interaction with Orai1-L2 (*p* = 0.008) than with Orai3-L2 (*p* = 0.02) (Fig. 5*d*). However, as the binding probability between truncated Orai1 NT and Orai3-L2 is very similar to that of non-truncated Orai1 NT and Orai1-L2 (*i.e.* ∼13%), we assume that this value might represent a critical threshold of interaction probability, above which inhibitory effects are likely to occur. In addition to the higher interaction frequency, the truncation mutant also showed significantly higher unbinding forces during dissociation from Orai1-L2 (*p* < 0.001), whereas no significant increase was observed for the interaction with Orai3-L2 (Fig. 5*e*). By varying the pulling velocity and plotting the most probable unbinding force as a function of the loading rate (*i.e.* the product of pulling velocity and effective spring constant), we further obtained information about the molecular transition during dissociation. In accordance with the model of Bell (54) and Evans and Ritchie (55), we observed a linear increase in unbinding forces with a logarithmically increasing loading rate (Fig. 5, *f* and *g*). Taking into account the S.D. value of the respective model fit, the determined dissociation rate *k*_off_ was not significantly changed upon truncation of Orai1 NT and interaction with Orai3-L2 (Fig. 5*e*), but it was about 10 times lower for the interaction with Orai1-L2 (Fig. 5*d*).

The combination of more frequent interactions, higher interaction forces, and slower dissociation rates thus indicated a stronger interplay between truncated Orai1-NT fragment and Orai1-L2, which might account for the inhibited, non-permissive channel conformations observed with N-terminal truncation of Orai1 wildtype and constitutively active Orai1 forms.

### Orai1 ΔN_1-78_ loses function due to an inhibitory interaction of Orai1-loop2 and the N terminus

**Figure 6.**
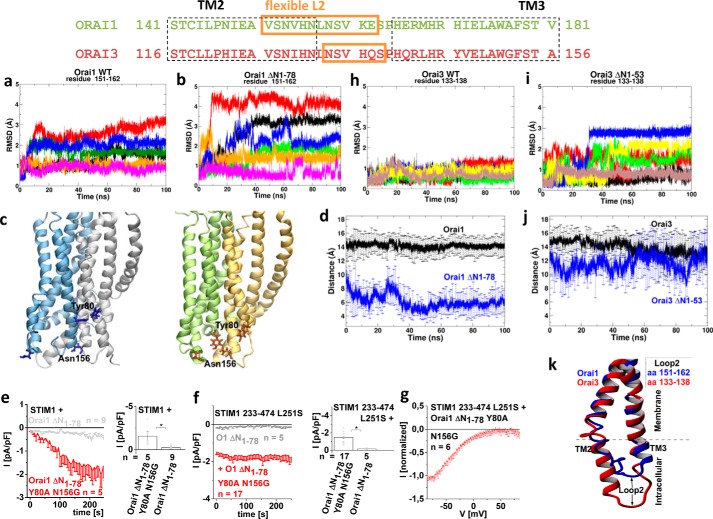
**Orai1 ΔN_1–78_ loses function due to an inhibitory interaction of Orai1-loop2 and the N terminus.** *a* and *b*, RMSD of flexible loop2-portion (aa 151–162) in Orai1 wildtype and Orai1 ΔN_1–78_ truncation mutant. RMSDs of different monomers (in total 6) are shown in *different colors. c*, *side view* of Orai1 full-length (*left*; two monomers are shown in *different colors*, and Tyr^80^ and Asn^156^ residues are shown as *blue sticks*) and Orai1 ΔN_1–78_ truncation mutant (*right*; two monomers are shown in *different colors*, and Tyr^80^ and Asn^156^ residues are shown *orange sticks*), showing interactions between loop2 and the N terminus of TM1. *d*, distance plot for Tyr^80^ (NT)–Asn^156^ (loop2) in Orai1 WT and ΔN_1–78_ truncation mutants calculated and averaged over all six monomers (*p* < 0.001 at *t* = 100 ns). *e*, time course of whole-cell inward currents at −74 mV activated by passive store depletion of HEK 293 cells co-expressing STIM1 + Orai1 ΔN_1–78_ Y80A N156G in comparison with STIM1 + Orai1 ΔN_1–78_ together with a block diagram showing maximum currents at *t* = 180 s. *f*, time course of whole-cell inward currents at −74 mV activated by passive store depletion of HEK 293 cells co-expressing STIM1 233–474 L251S + Orai1 ΔN_1–78_ Y80A/N156G in comparison with STIM1 233–474 L251S + Orai1 ΔN_1–78_ together with a block diagram showing maximum currents at *t* = 180 s (*, *p* < 0.05). *g*, *I*/V relationship of STIM1 233–474 L251S-mediated Orai1 ΔN_1–78_ Y80A/N156G currents. *h* and *i*, RMSD of flexible loop2 (aa 133–138) in Orai3 wildtype and Orai3 ΔN_1–53_ truncation mutant. RMSD of different monomers (in total 6) are shown in *different colors. j*, distance plot for Tyr^55^ (TM1)–Asn^131^ (loop2) in Orai3 WT and ΔN_1–53_ truncation mutants calculated and averaged over all six monomers. (*p* > 0.05 at *t* = 100 ns). *k*, comparison of *side views* of Orai1 (*blue*) and Orai3 (*red*) of TM2 and TM3 connecting the flexible loop2 portion in between after simulation of the human Orai1 and Orai3 models. On average, TM2 of Orai1 is shorter than that of Orai3. *, *p* < 0.05. *Error bars*, S.E.

To investigate how the Orai1-loop2 affects the structure of the non-functional N-truncation mutant *versus* wildtype Orai1, we performed molecular dynamics (MD) simulations of a dOrai-based 3D homology model of the hexameric human Orai1 channel (46) before and after N-truncation (for 100 ns each). Root mean square deviation (RMSD), hydrogen bond interaction, and distance analysis were performed on these trajectories. We discovered that, upon N-terminal deletion, the flexible portions of Orai1-loop2 regions (aa 151–162) of each monomer had more space to move apart from their initial positions in the full-length channel, as was visible via enhanced RMSD, and they settled into new positions within 80 ns (Fig. 6, *a* and *b*). Moreover, the initial residues Leu^79^ and Tyr^80^ in the truncated form were no longer stabilized by hydrogen bonds within the α-helix of the ETON region and altered their orientation (Fig. 6*c* and Fig. S4 (*a* and *b*)). In about five of six Orai1 monomers, the N-terminal residues interacted with residues of the loop2 (Leu^79^-Leu^157^/Ser^159^; Tyr^80^-Ser^152^/Asn^156^) of the same or adjacent subunit, exclusively in the truncated and not in the full-length form, as exemplarily shown for each pair of residues (Fig. 6 (*c* and *d*) and Fig. S4 (*a* and *b*)). These interactions were supposedly responsible for the non-permissive channel conformation impeding current activation of Orai1 ΔN_1–76/78_. Disruption of these interactions via single or double point mutations would probably recover Orai1 ΔN_1–78_ function. Whereas single point and several double mutations left Orai1 ΔN_1–78_ inactive (Fig. S4, *c* and *d*), the double mutant Orai1 ΔN_1–78_ Y80A/N156G indeed displayed partially restored activation of Ca^2+^-selective, inwardly rectifying currents in the presence of STIM1 as well as STIM1-OASF-L251S (Fig. 6, *e–g*). In agreement, the averaged distance for each time point calculated over all six Orai1 monomers suggested that the two residues Tyr^80^ and Asn^156^ were close enough to make contact in the N-terminal truncated Orai1. Thus, their point mutation probably disrupted their interaction (Fig. 6*d*). In full-length Orai1, these residues were far apart from each other and thus unlikely to form interactions (Fig. 6*d*), providing a permissive channel conformation for store-operated activation in contrast to the Orai1 N-truncation mutants. Control experiments revealed that full-length Orai1 remained unaffected by the double point mutation Y80S/N156G (Fig. S4*e*).

In an attempt to understand why analogue Orai3 N-truncation mutants remained functional, we analyzed a dOrai-based 3D homology model of the hexameric human Orai3 channel (Fig. S5) for comparison. Tyr^80^ and Asn^156^ were fully conserved in Orai3 (Tyr^55^ and Asn^133^). MD simulations of 100 ns, however, revealed that analogue residues were far apart for interaction (Fig. 6*j*) in Orai3 ΔN_1–53_ and full-length Orai3 (Fig. 6, *h* and *i*), in contrast to Orai1 ΔN_1–78_. The comparison of Orai1 and Orai3 structures upon simulation further displayed that especially TM2 helices are longer in Orai3 compared with Orai1, which suggested that the remaining flexible loop2 portion in between was shorter in Orai3 (aa 133–138) in contrast to Orai1 (aa 151–162) (see sequence alignment in Fig. 6 (*top*); Fig. 6*k*). These distinct structural properties might account for the isoform-specific functional effects of the Orai N-truncation mutants.

In summary, MD simulations and mutational analysis suggested that loss of function of Orai1 ΔN_1–78_ was mechanistically linked to inhibitory interactions of the Orai1-loop2 region with the truncated N-terminal portion. Such interactions did not occur in the full-length Orai channels or between the truncated N-terminal segment and Orai3-loop2. Disruption of this interaction partially restored function of the Orai1 ΔN_1–78_ truncation mutant in providing a permissive channel conformation for activation by STIM1.

### Cross-linking of the N terminus and loop2 in Orai1 leads to current inhibition

**Figure 7.**
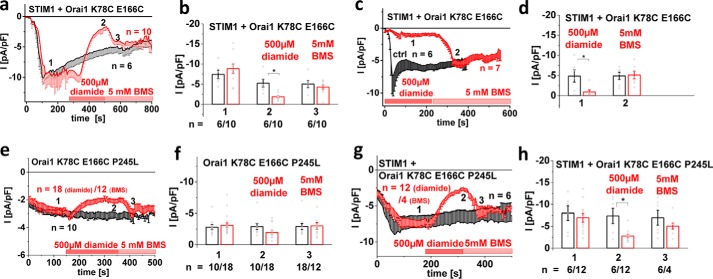
**Cysteine cross-linking of NT and loop2 of Orai1 reduces STIM1-mediated currents significantly.** Throughout the figure, *black* corresponds to untreated cells, and *red* corresponds to treated (with diamide/BMS) cells. *a*, time course of whole-cell inward currents at −74 mV activated upon passive store depletion of HEK 293 cells co-expressing STIM1 + Orai1 K78C/E166C. Upon maximal activation of STIM1-Orai1 K78C/E166C currents, cysteine cross-linking was induced via diamide (500 μm), and the subsequent release of disulfide bonds was induced via BMS (5 mm). *b*, bar graph comparing currents at the respective time points 1 (after maximal activation upon passive store depletion), 2 (after application of diamide), and 3 (after application of BMS). *c*, time course of whole-cell inward currents at −74 mV activated upon passive store depletion of HEK 293 cells co-expressing STIM1 + Orai1 K78C/E166C pretreated with diamide (500 μm). After 230 s, BMS (5 mm) was applied to release disulfide bonds. *d*, bar graph comparing currents at the respective time points 1 (after maximal activation upon passive store depletion in the presence of diamide) and 2 (after application of BMS). *e*, time course of whole-cell inward currents at −74 mV activated upon passive store depletion of HEK 293 cells expressing Orai1 K78C/E166C/P245L. Upon maximal activation of Orai1 K78C/E166C/P245L currents, cysteine cross-linking was induced via diamide (500 μm), and the subsequent release of disulfide bonds was induced via BMS (5 mm). *f*, bar graph comparing currents at the respective time points 1 (after maximal activation upon passive store depletion), 2 (after application of diamide), and 3 (after application of BMS). *g*, time course of whole-cell inward currents at −74 mV activated upon passive store depletion of HEK 293 cells co-expressing STIM1 + Orai1 K78C/E166C/P245L. Upon maximal activation of STIM1-Orai1 K78C/E166C/P245L currents, cysteine cross-linking was induced via diamide (500 μm), and the subsequent release of disulfide bonds was induced via BMS (5 mm). *h*, bar graph comparing currents at the respective time points 1 (after maximal activation upon passive store depletion), 2 (after application of diamide), and 3 (after application of BMS). *, *p* < 0.05; *error bars*, S.D.

MD simulations revealed a close proximity and interaction of N terminus and loop2 in the Orai1 ΔN_1–78_ mutant that was probably responsible for the loss of function. In an attempt to mimic structural arrangements in the non-functional Orai1 N-truncation mutants, we enforced interactions of the N terminus and loop2 in the full-length Orai1 channel by utilizing a cysteine-cross-linking approach. The crystal structure of dOrai1 revealed that Lys^78^ and Glu^166^ in hOrai1 were located quite close and assumedly formed salt bridges. Thus, both residues were mutated to cysteines and tested for their impact on Orai1 channel function upon chemical cross-linking via diamide and subsequent disruption of disulfide bonds via bis(2-mercaptoethyl)sulfone (BMS). For a control, single (K78C and E166C) and double substitutions (K78C/E166C) (Fig. S6) resulted in undisturbed STIM1-dependent, inwardly rectifying Ca^2+^ currents. Application of 500 μm diamide following full activation of Orai1 K78C/E166C resulted in substantially inhibited currents, which could again be enhanced upon perfusion of BMS (Fig. 7, *a* and *b*) to approximately the same level as under control conditions. Preapplication of diamide 1–2 min before establishing the whole-cell recording configuration led to substantially reduced currents from STIM1-dependent activation of Orai1 K78C/E166C, which were recovered by perfusion of BMS (Fig. 7, *c* and *d*). Control experiments confirmed that wildtype Orai1 as well as single point mutants Orai1 K78C and Orai1 E166C remained unaffected upon the addition of diamide or BMS (Fig. S6, *a–c*). Moreover, STIM1-mediated Orai1 K78C/E166C currents, activated to a maximal level, did not further increase subsequent to BMS application, suggesting that the cysteines were not natively cross-linked (Fig. S6*d*). Further, examination of two other cysteine double mutants, Orai1 K78C/H169C and Orai1 S82C/H169C (Fig. S6, *e* and *f*), that contained substitutions at nearby positions failed to evoke responses to diamide or BMS application, suggesting that the observed effects with Orai1 K78C/E166C were rather specific.

Additionally, we investigated the impact of cysteine cross-linking of the N terminus and loop2 on the function of the constitutively active mutant Orai1 P245L. Orai1 K78C E166C/P245L revealed current inhibition following application of diamide and restored current levels after perfusion of BMS (Fig. 7, *e–h*), both in the absence and in the presence of STIM1, which is in line with the experiments above.

In summary, chemical cross-linking of two residues, Lys^78^ in the Orai1 N terminus and Glu^166^ in loop2, resulted in current inhibition, which was reversed following BMS perfusion and disulfide disruption. Thus, cross-linking of Orai1 N terminus and loop2 impaired Orai1 activation, which might be mechanistically linked to the non-permissive channel conformation of Orai1 ΔN_1–78_, causing loss of function.

## Discussion

Our previously published studies (13, 38) on Orai N-truncation mutants have so far not clarified why only Orai1 ΔN_1–78_, and not the analogue Orai3 ΔN_1–53_, loses function, despite the fact that the remaining N-terminal portion is fully conserved among all of the Orai isoforms. Here we discovered that isoform-specific impairment of Orai function was not linked to the N terminus itself but to distinct isoform-specific properties of loop2. STIM1-dependent function of this Orai1 N-truncation mutant was restored upon the swap with Orai3-loop2. Loss of function of the Orai1 N-truncation mutant underlay inhibitory interactions of the N-terminal portion with Orai1-loop2. We suggest that the function of Orai channels is ensured by a fine-tuned, permissive interplay between the N terminus and loop2.

Several studies (8, 13, 41, 47, 48) have already demonstrated that the Orai1 N terminus, especially the conserved ETON region, plays an essential role in STIM1-mediated activation of Orai channels. As already reported (13), not a single N-terminal amino acid or hot spot, but several residues in the conserved N-terminal portion affect STIM1-mediated Orai activation. Here, we discovered that only residues in the second half of the ETON region (aa 80–90) were indispensable for Orai channel gating. In contrast, those in the first half (aa 73–79) were not necessarily required but predominantly supported STIM1-dependent Orai1 activation, as long as the Orai1-loop2 did not act in an inhibitory manner. MD simulations in line with our atomic force microscopy studies suggested that isoform-specific structural properties of Orai1-loop2 led to inhibitory interactions within N-truncated Orai, thereby establishing a non-permissive channel conformation and impaired gating. The first half of the ETON region, in providing a permissive interplay with Orai3-loop2, not only supported STIM1-dependent Orai activation but also maintained typical CRAC channel hallmarks, thus fine-tuning Orai channel function (71).

Co-localization of STIM1 cytosolic fragments with the functional N-truncated Orai1 Orai3-loop2 chimera was enhanced but did not reach levels obtained with wildtype Orai1, which suggested that the N-terminal residues in the first half of the ETON region also supported STIM1 coupling, either in a direct or allosteric manner. This increased coupling of STIM1 with the N-truncated Orai1 Orai3-loop2 chimera was in line with restored selectivity in the presence of STIM1 when V102A was introduced, suggesting that STIM1 coupled more efficiently in the presence of Orai3-loop2 than Orai1-loop2. Park *et al.* (41) discovered no interaction of CRAC activation domain and an Orai1-loop2 fragment by co-immunoprecipitation. In contrast, by employing a longer loop2 fragment and the more specific FIRE system (42), we identified *in vitro* coupling of a STIM1 C-terminal fragment to loop2 fragments of both isoforms to a comparable extent. Constitutive mutants revealed that the loop2 effect already occurred at the level of Orai in the absence of STIM1. The constitutive Orai1 activity, lost upon N-truncation, was restored by the swap of Orai3-loop2, suggesting that regained function via Orai3-loop2 primarily underlay an altered conformation of the N-truncated Orai isoforms. We propose that in the N-truncated Orai1, the Orai1-loop2 has space to move toward the pore and forms interactions with the truncated N terminus. Indeed, the function of Orai1 ΔN_1–78_ was recovered upon the break of an interaction between the truncated N terminus and Orai1-loop2, as derived from MD simulations. In contrast to Orai1 N-truncation mutants, analogous N-terminal truncations of Orai3 remained functional. A comparison of Orai1 and Orai3 in MD simulations indicated that the helical portion of TM2 extending into the cytosol might be shorter in Orai1. The latter suggested a longer, flexible loop2 portion in Orai1 compared with Orai3 that potentially possessed a higher degree of freedom, thus coming closer to the N-truncated Orai segments to form interactions. The close positioning of the truncated N-terminal portion and Orai1-loop2 probably impaired gating, inducing a non-permissive channel conformation and loss of Orai activation. Thus, Orai proteins require fine-tuned, permissive communication between the N terminus and loop2 for maintenance of store-operated function.

Orai1-loop2 has already been reported (49) to function in an inhibitory manner, as the conserved amino acid stretch ^151^VSNV^154^ especially contributes to fast Ca^2+^-dependent inactivation, probably acting as a blocking particle or impacting inactivation in an allosteric manner.

In addition to the inhibitory effect of Orai1-loop2 on the N terminus, we inferred that it impaired STIM1 binding as well. STIM1 coupling was restored upon the swap of Orai3-loop2 to the N-truncated Orai1 mutant. However, it remained unclear whether the remaining N terminus or loop2 or even both contributed directly or allosterically to STIM1 coupling. Interaction studies on STIM1 and Orai1 fragments revealed that an interaction between the Orai1 N terminus and STIM1 C terminus is probable (13, 41). In this study, we were further able to show interactions between fragments of STIM1-C terminus and Orai-loop2. We suggest that the predicted coupling of the truncated N-terminal region with loop2 of Orai1 masks STIM1-coupling sites, either in the N terminus, loop2, or both. Preserved function of analogue Orai3 N-truncation mutants suggested that this isoform lacked the inhibitory interactions of the remaining N terminus and loop2, in line with MD simulations suggesting a shorter flexible loop2 portion of Orai3. Thus, potential STIM1-binding sites remained accessible in Orai3 N-truncation mutants. Altogether, our chimeric approaches revealed that loop2 contributed to overall STIM1 binding. However, additional investigations are required to establish whether the N terminus, the loop2, or both together contribute to STIM1 coupling, in addition to the Orai C terminus, which represents the main STIM1-binding site.

The distinct structure of Orai1-loop2 and Orai3-loop2, including the helical portion of the cytosolic TM2 and TM3 extensions as well as a flexible linker in between, seemed to be determined by a few non-conserved residues. We discovered that the swap of five residues from Orai3-loop2 into Orai1-loop2 was required and sufficient for restoring STIM1-dependent activation of the Orai1 N-truncation mutants. In agreement, MD simulations suggested that the cytosolic helical extension of TM2 is longer in Orai3 than in Orai1, thus making the flexible portion in between shorter in Orai3 compared with Orai1. Hence, the overall structure of the cytosolic stretch connecting TM2 and TM3 probably determined the function of Orai N-truncation mutants.

In contrast to the Orai1 and Orai3 N-truncation mutants, the full-length channels did not feature such drastic differences in function depending on the loop2, although MD simulations suggested distinct structural properties of the loop2 regions connecting TM2 and TM3. In support, predicted interacting residues between the N terminus and loop2 (Tyr^80^ and Asn^156^) in the truncated Orai1 mutants were farther apart from each other in the full-length Orai1 channel. This difference might be caused by an altered orientation of residues in the truncated N-terminal portion, in contrast to that in the full-length Orai1. Specifically, Leu^79^ and Tyr^80^ were no longer stabilized by hydrogen bonds within the α-helix of the ETON region in the truncated form. Cysteine-cross-linking studies revealed that an enforced interaction of residues in the N terminus and loop2, mimicking the conformational arrangement in the Orai1 N-truncation mutant, resulted in current inhibition, which could be reversed by disulfide bond disruption, likewise independent of STIM1. Hence, full-length Orai channels feature permissive communication of the N terminus and loop2, ensuring store-operated activation.

In conclusion, isoform-specific requirements of Orai1 and Orai3 with respect to maintenance of channel function of the N-truncated forms did not underlie altered properties of the N terminus, but were rather caused by the distinct structural behavior of the loop2 of Orai1 and Orai3. We suggest that maintenance of store-operated activation of wildtype Orai proteins is ensured by fine-tuned, permissive communication of the N terminus and the loop2 region.

## Experimental procedures

### Molecular biology

Human STIM1 (STIM1; accession number NM_003156) N-terminally ECFP-tagged was kindly provided by the laboratory of T. Meyer (Stanford University). STIM1 C terminus (aa 233–685) was cloned into the T/A site of pcDNA3.1V5 His TOPO by PCR and subcloned into pECFP-C1 via KpnI and XbaI. pECFP-C1 STIM1 C terminus was used as template for the generation of pECFP-OASF by introducing a stop codon at position 475 (aa 233–474). pECFP-STIM1 and pECFP-OASF were used as templates for the generation of pECFP-STIM1 L251S and pECFP-OASF L251S, respectively. All point mutations were performed using the QuikChange XL site-directed mutagenesis kit (Stratagene).

For N-terminal fluorescence labeling of human Orai1 (Orai1; accession number NM_032790, provided by the laboratory of A. Rao) as well as human Orai3 (Orai3; accession number NM_152288, provided by the laboratory of L. Birnbaumer), the constructs were cloned into the pEYFP-C1 (Clontech) expression vector via KpnI/XbaI (Orai1) and BamHI/XbaI (Orai3) restriction sites, respectively.

Orai1 N-terminal deletion mutants (Orai1 ΔN_1–76_, ΔN_1–78_, ΔN_1–80_, ΔN_1–82_) were amplified via PCR, including an N-terminal KpnI and a C-terminal XbaI restriction site; Orai3 N-terminal deletion mutants (Orai3 ΔN_1–51_, ΔN_1–53,_ ΔN_1–55_, ΔN_1–57,_ and ΔN_1–60_) were amplified via PCR, including an N-terminal BamHI and a C-terminal XbaI restriction site for cloning into the pEYFP-C1 vector. Chimeric constructs (Orai1-Orai3-loop2; Orai1 ΔN_1–76_; Orai1 ΔN_1–78_-, ΔN_1–80_-, and ΔN_1–82_-Orai3-loop2, respectively; Orai1 ΔN_1–76_- and ΔN_1–78_-Orai3-C-term, respectively; Orai1 ΔN_1–78_-Orai3-loop2-C-term; Orai1 ΔN_1–78_-Orai3-TM3-C-term; Orai1 ΔN_1–78_-Orai3-TM4-C-term; and Orai3 ΔN_1–53_-Orai1-loop2) were cloned via SOEing (Splicing by Overlap Extension) into the pEYFP-C1 (Clontech) expression vector for N-terminal fluorescence labeling. Site-directed mutagenesis (N147H, H171Y, K161H/E162Q/E166Q, N147H/K161H/E162Q/E166Q/H171Y, N147H/V154I/K161H/E162Q/H171Y, I144L/N147H/K161H/E162Q/E166Q/H171Y, V102A, V77A, L74E/W76E, K77A/R78A, L81A/S82A, K85E, P245L, L185A, V181A, L74E/W76E/P245L, K85E/P245L, K78C/E166C, K78C/E166C/P245L, Y80A/N156G, L79S, Y80S, Y80G, R83A, R83S, L79S/S159S, L79A/S159G, L79S/S159G, L79S/S159A, Y80A/S156G, Y80G/S156A, and Y80G/S156G) was performed using the QuikChange^TM^ XL site-directed mutagenesis kit (Stratagene) with the corresponding Orai1 and/or Orai1-Orai3 chimeric constructs serving as templates. The same procedure was used for Orai3 Δ1–53/Δ1–57 P254L and F160A.

YFP- and CFP-labeled FIRE constructs (TMG-Orai1-loop2-aa 137–184 and TMG-Orai3-loop2-aa 112–159, TMG-STIM1_388–430) were cloned according to Fahrner *et al.* (42).

### Supplemental constructs

Supplemental constructs were as follows: pEYFP-C1 Orai3 Δ_1–53_ Orai1-L2–141-181; pEYFP-C1 Orai3 Δ_1–53_ Orai1-L2–144-172; pEYFP-C1 Orai1 Δ_1–78_ Orai3-L2–116-155-CT; pEYFP-C1 Orai1 Δ_1–78_ Orai3-L2–116-155; pEYFP-C1 Orai1 Δ_1–78_ Orai3-L2–119-147; pEYFP-C1 Orai1 Δ_1–78_ Orai3-L2–136-141; pEYFP-C1 Orai1 Δ_1–78_ Orai3-TM3-CT; and pEYFP-C1 Orai1 Δ_1–78_ Orai3-TM4-CT. Site-directed mutagenesis (N147H, K161H/E162Q/E166Q, K161H/E162Q/E166Q/H171Y, N147H/K161H/E162Q/E166Q/H171Y, H171Y, L79S, Y80S, Y80G, R83A, R83S, N158A, N158G, S159A, S159G, L79A/S159A, L79A/S159G, L79S/S159G, L79S/S159A, Y80A/S156G, Y80G/S156A, Y80G/S156G, N156A/N159A, K78C, E166C, K78C/E166C, K78C/E169C, and S82C/H169C) was performed using the QuikChange^TM^ XL site-directed mutagenesis kit (Stratagene) with the corresponding Orai1 and/or Orai1-Orai3 chimeric constructs serving as templates. All constructs were confirmed by sequence analysis.

### Cell culture and transfection

Transient transfection of HEK 293 cells was performed (50) using either the TransFectin lipid reagent (Bio-Rad) or the TransPass transfection reagent (New England Biolabs). The CRISP/Cas9 STIM1 knockout HEK cells were kindly provided by M. Trebak (Penn State).

### Electrophysiology

Electrophysiological recordings comparing characteristics of 2–3 constructs were carried out in paired comparison on the same day. Expression patterns and levels of the various constructs were carefully monitored by confocal fluorescence microscopy and were not significantly changed by the introduced mutations. Electrophysiological experiments were performed at 20–24 °C, using the patch-clamp technique in the whole-cell recording configuration. For STIM1/Orai as well as STIM1 C terminus/Orai current measurements, voltage ramps were usually applied every 5 s from a holding potential of 0 mV, covering a range of −90 to +90 mV over 1 s. The internal pipette solution for passive store depletion contained 3.5 mm MgCl_2_, 145 mm cesium methane sulfonate, 8 mm NaCl, 10 mm HEPES, 20 mm EGTA, pH 7.2. The 100 nm Ca^2+^-containing intracellular solution included 3.5 mm MgCl_2_, 145 mm cesium methane sulfonate, 8 mm NaCl, 10 mm HEPES, 10 mm EGTA, 4.3 mm CaCl_2_, pH 7.2. The extracellular solution consisted of 145 mm NaCl, 5 mm CsCl, 1 mm MgCl_2_, 10 mm HEPES, 10 mm glucose, 10 mm CaCl_2_, pH 7.4. All currents were leak-corrected by subtraction of the leak current remaining following 10 μm La^3+^ application.

Chemical cross-linking was obtained via diamide, whereas disruption of disulfide bonds was obtained via BMS. Diamide was dissolved in water, and the stock solution contained 1 m. To achieve the final concentration of 500 μm, the diamide stock was further dissolved in the extracellular solution. BMS was dissolved in the extracellular solution to a final concentration of 5 mm.

### Confocal fluorescence microscopy

Confocal microscopy for co-localization experiments was performed in a manner similar to that described previously (51). In brief, a QLC100 real-time confocal system (VisiTech International, Sunderland, UK) was used for recording fluorescence images connected to two Photometrics CoolSNAPHQ monochrome cameras (Roper Scientific) and a dual port adapter (dichroic: 505lp; cyan emission filter: 485/30; yellow emission filter: 535/50; Chroma Technology Corp.). This system was attached to an Axiovert 200M microscope (Zeiss, Jena, Germany) in conjunction with an argon ion multiwavelength (457, 488, and 514 nm) laser (Spectra Physics). The wavelengths were selected by an Acousto Optical tunable filter (VisiTech International). MetaMorph version 5.0 software (Universal Imaging Corp.) was used to acquire images and to control the confocal system. Illumination times for CFP and YFP images that were consecutively recorded with a minimum delay were about 900 ms.

### AFM single-molecule force spectroscopy

Orai1-L2 (aa 136–186, mutation C143G) and Orai3-L2 (aa 111–161, mutation C118G) were custom-synthesized with >95% purity by Synpeptide. Orai1 N-terminal fragments aa 70–91 and 79–91 were custom-synthesized with a C-terminal cysteine for site-specific coupling with >95% purity by DgPeptides. Orai1-L2 and Orai3-L2 were conjugated to amino-functionalized AFM cantilever tips (Si_3_N_4_/MSCT, Bruker) via a flexible NHS-PEG18-acetal cross-linker (44, 45). Similarly, Orai1-NT 70–91 and Orai1-NT 79–91 were immobilized on silicon chips via NHS-PEG27-maleimide linkers.

All force measurements were carried out at room temperature in HBS buffer (150 mm NaCl, 5 mm Hepes) by using a PicoPlus 5500 atomic force microscope (Agilent Technologies) and cantilevers with a nominal spring constant of 0.03 newtons/m (MSCT, Bruker). Exact spring constants were determined using the thermal noise method (52). The functionalized cantilever was moved toward the surface until a certain deflection of the cantilever (*i.e.* force limit) was reached. Cantilever deflection was continuously observed by a laser beam focused on the cantilever back side and plotted over the tip-surface distance. Deflection (*z*) was converted to corresponding force values (*F*) according to Hooke's law (*F* = *k* × Δ*z*, where *k* represents the cantilever spring constant). Upon interaction, a pulling force was developed during the upward movement of the cantilever. At a critical force (*i.e.* unbinding force), the formed complex dissociated, and the cantilever jumped back into its neutral position. At least 1000 force–distance curves with three functionalized tips were performed, and the average binding probability was calculated. Binding probability was defined as the fraction of force curves showing a specific unbinding event. Specificity of the interaction was proofed by control experiments using tips that were only carrying the PEG linker. Dynamic force spectroscopy measurements were performed by varying the force rate with which the molecular bond is loaded (*i.e.* loading rate, product of pulling velocity and effective spring constant (*k*_eff_)). For this, the pulling velocity was varied between 50 and 8000 nm/s, resulting in loading rates from ∼10^2^ to ∼10^5^ pN/s. Force curves were analyzed as described by Baumgartner *et al.* (53). Unbinding events were identified during cantilever retraction by a typical non-linear increase in force due to stretching of the elastic PEG linker, followed by an abrupt return (jump) to the baseline. The height of this jump directly reflects the unbinding (rupture) force, and the slope at the time of rupture represents the spring constant of the PEG linker, *k*_PEG_. The effective spring constant of the system, *k*_eff_, was calculated according to the equation, *k*_eff_ = (*k*_PEG_^−1^ + *k_c_*^−1^)^−1^, where *k_c_* represents the cantilever spring constant as determined by the thermal noise method. The loading rate of each individual force–distance curve was calculated by multiplying the effective spring constant with the pulling velocity. The probability density function (pdf) of all unbinding events per pulling velocity was constructed as an empirical estimate for the force distribution, and the most probable unbinding force was determined by fitting a Gaussian distribution to the pdf. According to the single energy barrier model (54, 55), the most probable unbinding force (F*) is given as a function of the loading rate (*r*),

(1) $$F*=\frac{k_{B}T}{x_{\beta }}\;\text{In}\;\left(\frac{x_{\beta }}{k_{B}T}\frac{r}{k_{\text{off}}}\right)$$

where *k_B_T* is the thermal energy, *x*_β_ is the thermally averaged projection of the transition state along the force direction (width of energy barrier), and *k*_off_ is the dissociation rate constant. Based on the theory that a single energy barrier is crossed in the thermally activated regime, the most probable unbinding force is expected to rise linearly with a logarithmically increasing loading rate. The parameters *x*_β_ and *k*_off_ that characterize the molecular transition during dissociation were determined by fitting F* against ln *r*.

### MD simulations

The 3D coordinates published (46) and deposited in the Model Archive database (https://www.modelarchive.org/doi,^5^ DOI identifier: 10.5452/ma-akdjp) were used for MD simulations of human Orai1. For preparation of a full atomistic model of human Orai3 (Fig. S5), the *D. melanogaster* Orai1 crystal structure (Protein Data Bank code 4HKR) was used as a template. The sequence identity of *D. melanogaster* with human Orai3 is 56% with a sequence similarity of 68%. The macrohm_built.mcr script in YASARA was applied to perform the homology modeling, and 3D coordinates of conserved residues were restrained to the values in the crystal structure using FixModelRes option (56) during model building. The Orai3 model has a *Z*-score of −2.109 as calculated by YASARA, and 93.5% of the residues are in the most favored region of the Ramachandran plot with a *G*-factor of 0.11 calculated by PROCHECK (57).

The human Orai1/Orai3 models were embedded in a pre-equilibrated POPC membrane using the inflategro method (58) in GROMACS version 4.6.5 (59, 60). WHATIF implemented in YASARA was used to predict the protonation states of histidines (61). Berger parameters for lipids (62) were converted into OPLS (63) format according to the method proposed by Neale to describe POPC molecules. The membrane-protein system was solvated into a simple point charge water model (63), and the system was further neutralized by adding Na^+^ and Cl^−^ ions. The final concentration of 10 mm CaCl_2_ and 8 mm NaCl was achieved by adding Ca^2+^, Na^+^, and Cl^−^ ions. The MD simulations were performed in an isothermal-isobaric (NPT) ensemble at 310 K temperature using a time step of 2 fs. The velocity rescale thermostat (64) and Parrinello–Rahman barostat (65) were used to maintain the temperature at 310 K and pressure at 1 bar during early equilibration steps with position restraints on protein and lipid atoms. Position restraints were gradually decreased, and final MD simulations were performed with no position restraints. Nose–Hoover thermostat (66, 67) and Parrinello–Rahman barostat were used during this final run. The LINCS algorithm was used to constrain bond lengths (68). The particle mesh Ewald method was used to calculate long-range electrostatic interactions with a cutoff distance of 10.0 Å (69). The Lennard-Jones 6–12 potential was used to calculate van der Waals interactions with a 10-Å cutoff distance. VMD (70) and GROMACS version 4.6.5 were used for the analysis of simulation trajectories. The Xmgrace tool (http://plasmagate.weizmann.ac.il/Grace/)^4^ was used for preparing the graphs.

### Statistics

Results are presented as means ± S.E. calculated for the indicated number of experiments. Student's two-tailed *t* test was used for statistical comparison, considering differences statistically significant at *p* < 0.05.

## Author contributions

I. D. and C. R. conceived and coordinated the study and wrote the paper. I. D., C. B., M. S., A. K., and R. S. performed and analyzed electrophysiological experiments. M. M. carried out fluorescence microscopy experiments. M. S., M. F., C. B., P. P., and I. F. contributed to molecular biology. L. T., H. G., and P. H. carried out and analyzed AFM measurements. S. K. P., V. Z., and R. E. conducted and analyzed MD simulation. All authors reviewed the results and approved the final version of the manuscript.

## Supplementary Material

Supporting Information

## Footnotes

5 Please note that the JBC is not responsible for the long-term archiving and maintenance of this site or any other third party hosted site.

## Footnotes

4 The abbreviations used are:

CRAC: Ca^2+^ release-activated Ca^2+^
STIM1: stromal interaction molecule 1
TM: transmembrane domain
ETON: extended transmembrane Orai N terminus
dOrai: *D. melanogaster* Orai
CAR: Ca^2+^-accumulating region
FIRE: FRET-derived interaction in a restricted environment
aa: amino acids
NT: N terminus
L2: loop2
AFM: atomic force microscopy
MD: molecular dynamics
RMSD: root mean square deviation
BMS: bis(2-mercaptoethyl)sulfone
CFP: cyan fluorescent protein
YFP: yellow fluorescent protein
pN: piconewtons
pdf: probability density function
POPC: 1-palmitoyl-2-oleoyl-*sn*-glycero-3-phosphocholine.
