# Supplementary material for: Communication between N terminus and loop2 tunes Orai activation
Source: J Biol Chem. 2017 Dec 13;293(4):1271–85. doi: 10.1074/jbc.M117.812693 (PMC5787804; doi:10.1074/jbc.M117.812693)
Supplement: Supporting Information [file supp_293_4_1271__index.html]

Communication between N-terminus and Loop2 tunes Orai activation — Communication between N terminus and loop2 tunes Orai activation — Communication between N terminus and loop2 tunes Orai activation — Supporting Information 

# Communication between N terminus and loop2 tunes Orai activation

## Supporting Information

- Suppl Figs. 1-6 (.pdf, 1.4 MB) - Suppl Figs. 1-6 including legends
