# Supplementary material for: Communication between N terminus and loop2 tunes Orai activation
Source: J Biol Chem. 2017 Dec 13;293(4):1271–85. doi: 10.1074/jbc.M117.812693 (PMC5787804; doi:10.1074/jbc.M117.812693)

Supplementary Figure 1: Orai1 truncation mutants require more of the ETON region than Orai3 for preserved store-operated activation

a) b) Schematic depiction of Orai1 ΔN$_{1-76}$ and Orai1 ΔN$_{1-78}$ highlighting functional properties in comparison to the corresponding Orai3 mutants (Orai3 ΔN$_{1-51}$ and Orai3 ΔN$_{1-53}$).

Supplementary Figure 2: Non-functional Orai1 N-truncation mutants regain significant function upon the swap of Orai3-L2.

Sequence alignment of Orai1- and Orai3-L2 depicting loop2-fragment used in the chimeras (framed orange, green and red) a) Time-course of whole cell inward currents at -74 mV activated by passive store-depletion of HEK 293 cells co-expressing STIM1 + Orai1 ΔN$_{1-76/78}$ or STIM1 + Orai1 ΔN$_{1-76/78}$ Orai3-C-term or STIM1 + Orai1 ΔN$_{1-76}$ Orai3-loop2. b) Block diagram showing STIM1-mediated maximal currents at t = 200 s upon whole cell break-in of STIM1-mediated Orai1 ΔN$_{1-76/78}$, Orai1 ΔN$_{1-76/78}$ Orai3-C-term and Orai1 ΔN$_{1-76}$ Orai3-loop2 currents in comparison. c) Block diagram showing STIM1-mediated maximal currents at t = 200 s upon whole cell break-in of Orai1 in comparison to Orai1 ΔN$_{1-78}$, Orai1 ΔN$_{1-78}$ Orai3-TM4-C-term, Orai1 ΔN$_{1-78}$ Orai3-TM3-C-term, Orai1 ΔN$_{1-78}$ Orai3-L2-136-141, Orai1 ΔN$_{1-78}$ Orai3-L2-119-147, Orai1 ΔN$_{1-78}$ Orai3-L2-116-156, Orai1 ΔN$_{1-78}$ Orai3-L2-116-156-C-term. d) Block diagram showing STIM1-mediated maximal currents at t = 200 s upon whole cell break-in of Orai3 in comparison to Orai3 ΔN$_{1-53}$, Orai3 ΔN$_{1-53}$ Orai1-L2-144-172, Orai3 ΔN$_{1-53}$ Orai1-L2-141-181. e) Time-course of constitutive whole cell inward currents at -74 mV activated via STIM1-OASF (233-474) of Orai1 in comparison to Orai1 ΔN$_{1-78}$ and Orai1 ΔN$_{1-78}$ Orai3-loop2. f) Block diagram showing STIM1-OASF-mediated maximal currents at t = 0 s upon whole cell break-in of Orai1 in comparison to Orai1 ΔN$_{1-78}$, Orai1 ΔN$_{1-78}$ Orai3-loop2 and Orai3 in comparison to Orai3 ΔN$_{1-53}$ and Orai3 ΔN$_{1-53}$ Orai1-L2. g) Block diagram showing STIM1-OASF-L251S-mediated maximal currents at t = 0 s upon whole cell break-in of Orai1 in comparison to Orai1 ΔN$_{1-78}$, Orai1 ΔN$_{1-78}$ Orai3-loop2 and Orai3 in comparison to Orai3 ΔN$_{1-53}$ and Orai3 ΔN$_{1-53}$ Orai1-L2. h) Time-course of whole cell inward currents at -74 mV activated by passive store-depletion of CRISP/Cas9 STIM1-KO HEK 293 cells co-expressing STIM1 + Orai1, STIM1 + Orai1 ΔN$_{1-78}$ or STIM1 + Orai1 ΔN$_{1-78}$ Orai3-loop2. i) Time-course of constitutive whole cell inward currents at -74 mV activated via STIM1-OASF (233-474) L251S of Orai1 ΔN$_{1-78}$ Orai3-C-term including the point mutations N147H, H171Y, K161H E162Q E166Q (3x) or N147H K161H E162Q E166Q H171Y (5x). j) Block diagram showing STIM1-OASF-L251S-mediated maximal currents at t = 0 s upon whole cell

break-in of Orai1 ΔN_{1-78} Orai3-C-term including the point mutations N147H, H171Y, K161H E162Q E166Q (3x) or N147H K161H E162Q E166Q H171Y (5x). j)

Supplementary Figure 3: Non-functional Orai3 truncation mutants display no rightward shift in the reversal potential upon additional introduction of V77A in the presence of STIM1.

a) I/V relationships of Orai3 $\Delta N_{1-51/53/55/57}$ V77A currents. b) I/V relationships of STIM1 + Orai3 $\Delta N_{1-51/53/55/57}$ V77A currents. c) Block diagram displaying reversal potentials of Orai3 $\Delta N_{1-51/53/55/57}$ V77A in the presence and absence of STIM1.

Supplementary Figure 4: Interactions formed between the remaining N-terminal segment and loop2 of Orai1 ΔN_{1-78}

a) Interaction of TM1 Tyr80 and loop2 Asn156 (shown as sticks) (left) Distance plot showing interactions Tyr80-Asn156 (in black) and Tyr80-Ser152 (in red) (right). In all cartoon representations loop2 is shown in blue, TM1 in magenta and other secondary structures are colored in green. b) Interaction TM1 N-terminal Leu79 (amino group) and backbone oxygen of Ser159 of loop2 (top left). Interaction TM1 N-terminal Leu79 (amino group) and backbone oxygen of Leu157 of loop2 (bottom left). Channel is shown upside down. Time distance plot for TM1 Leu79 - Ser 159 interaction (in red) and for TM1 Leu79 - Leu157 interaction (in black) (right). c) Block diagram showing STIM1-OASF-L251S-mediated maximal currents at t = 0 s upon whole cell break-in of Orai1 in comparison to Orai1 ΔN_{1-78} including the following single point mutations: L79S, Y80S, Y80G, S159A, S159G. d) Block diagram showing STIM1-OASF-L251S-mediated maximal currents at t = 0 s upon whole cell break-in of Orai1 in comparison to Orai1 ΔN_{1-78} including the following double point mutations: L79A S159A, L79A S159G, L79S S159G, L79S S159A, Y80A N156G, Y80G N156A, Y80G N156G. e) Time-course of whole cell inward currents at -74 mV activated by passive store-depletion of HEK 293 cells co-expressing STIM1 + Orai1 compared to STIM1 + Orai1 Y80A N156G.

Supplementary Figure 5: Atomic 3D model of human Orai3 (aa Gln43 to Val295), including the loop segments that were not present in the Drosophila Orai crystal structure on which this human Orai3 model is based. The panel shows a side view of a modelled hexameric structure with each monomer individually colored.

Supplementary Figure 6: Cysteine crosslinking of NT and L2 of Orai1. a) Time course of whole cell inward currents at -74 mV activated upon passive store-depletion of HEK 293 cells co-expressing STIM1 + Orai1. Upon maximal activation of STIM1 – Orai1 currents, diamide (500µM) and subsequently BMS (5mM) were applied. b) Time course of whole cell inward currents at -74 mV activated upon passive store-depletion of HEK 293 cells co-expressing STIM1 + Orai1 K78C or Orai1 E166C pretreated with 500µM diamide. c) Time course of whole cell inward currents at -74 mV activated upon passive store-depletion of HEK 293 cells co-expressing STIM1 + Orai1 K78C, Orai1 E166C or Orai1 K78C E166C. Upon maximal activation of STIM1 – Orai1 mutant currents, diamide (500µM) were applied. d) Time course of whole cell inward currents at -74 mV activated upon passive store-depletion of HEK 293 cells co-expressing STIM1 + Orai1 K78C E166C. Upon maximal activation of STIM1 – Orai1 K78C E166C currents, BMS (5mM) was applied. e) f) Time course of whole cell inward currents at -74 mV activated upon passive store-depletion of HEK 293 cells co-expressing STIM1 + Orai1 K78C H169C and Orai1 S82C H169C. Upon maximal activation of STIM1 – Orai1 currents, diamide (500µM) and subsequently BMS (5mM) (e) or just diamide (f) were applied.

Supplementary Figure 1

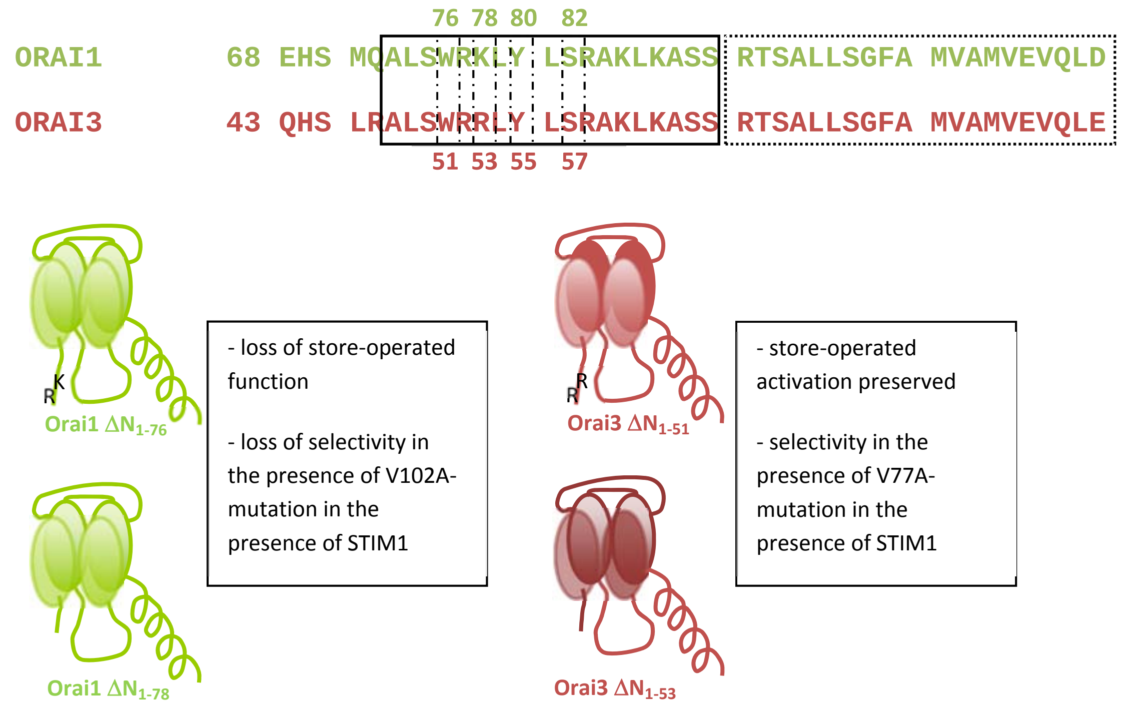

[1] Bergsmann, J., et al., *Molecular determinants within N terminus of Orai3 protein that control channel activation and gating.* J Biol Chem, 2011. 286(36): p. 31565-75.

[2] Derler, I., et al., *The extended transmembrane Orai1 N-terminal (ETON) region combines binding interface and gate for Orai1 activation by STIM1,* J Biol Chem, 2013. 288(40): p. 29025-34.

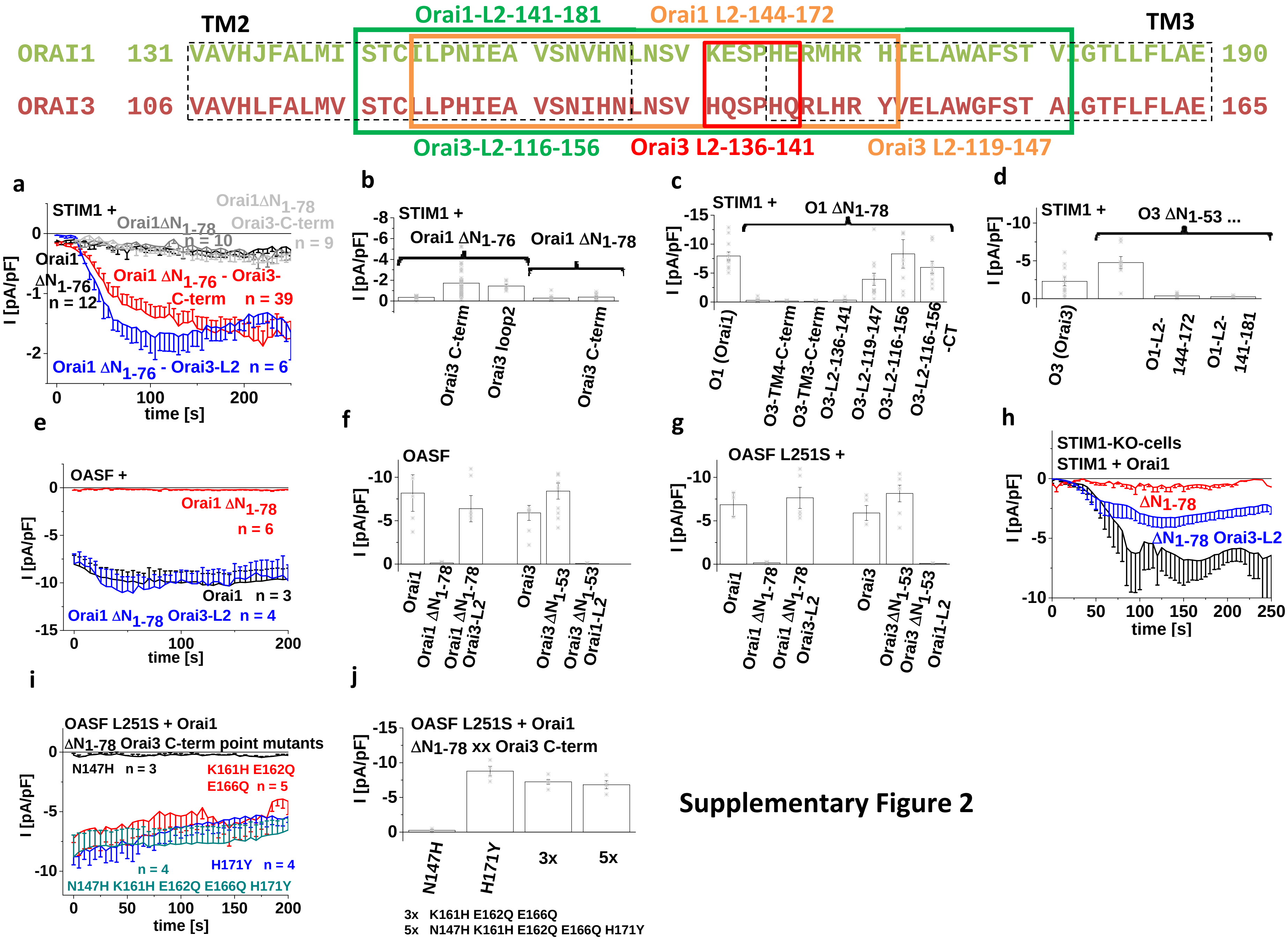

Supplementary Figure 2

Supplementary Figure 3

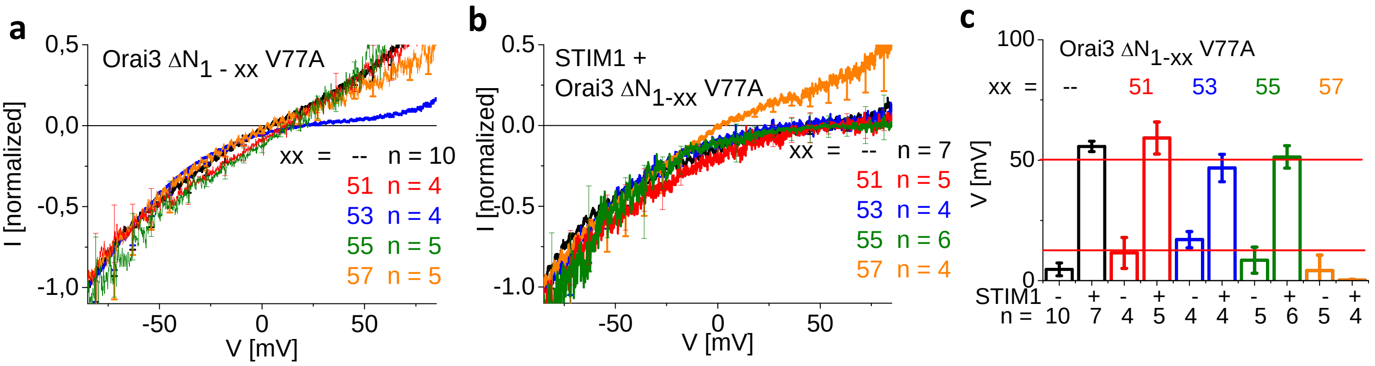

Supplementary Figure 4

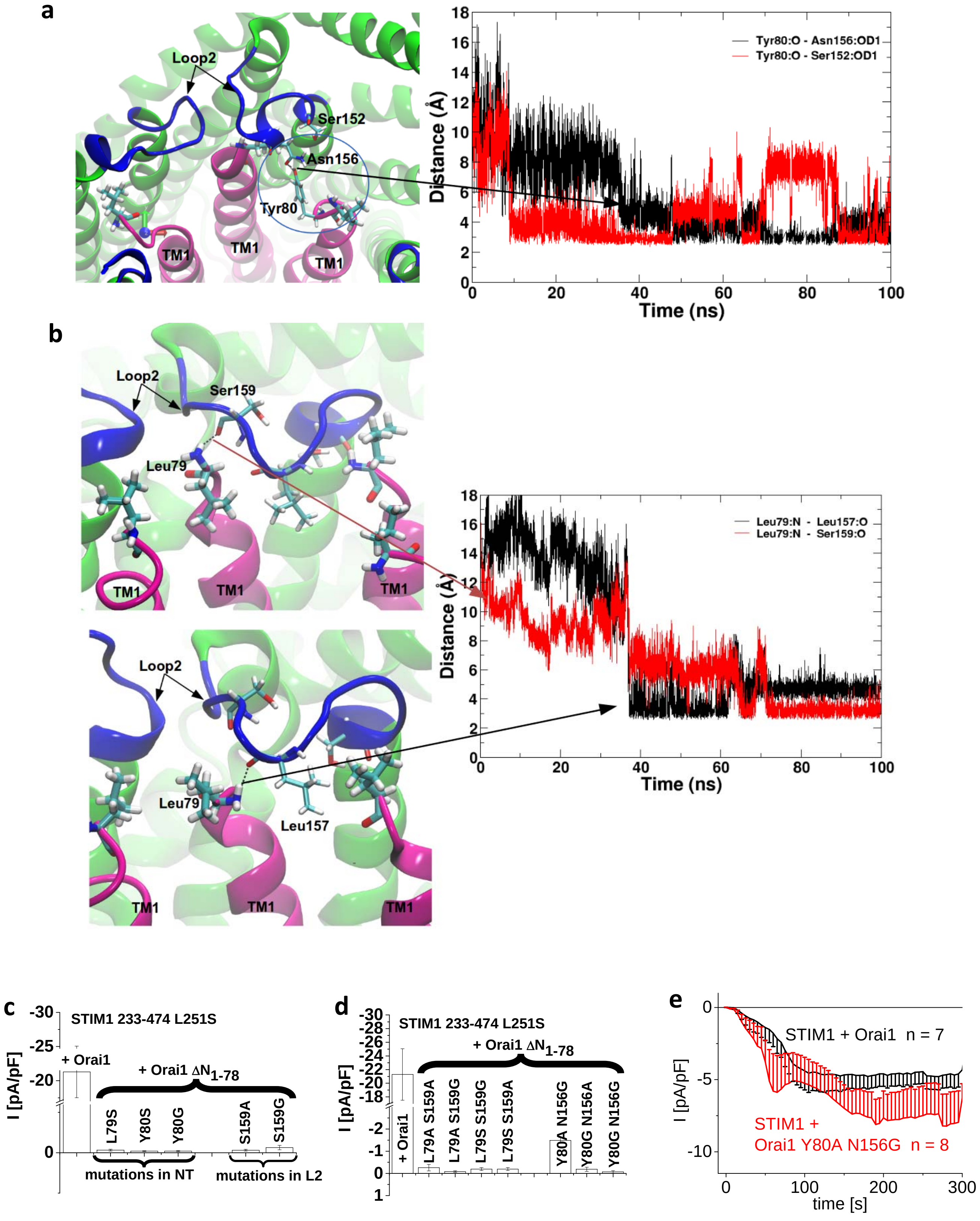

Supplementary Figure 5

3D model of human Orai3

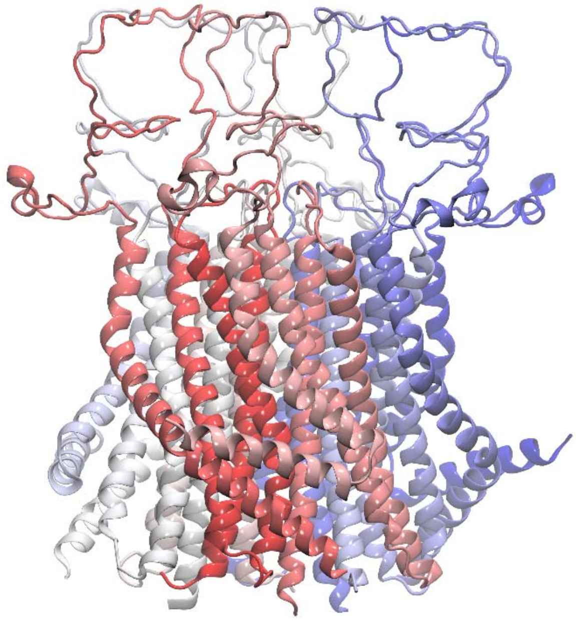

Supplementary Figure 6

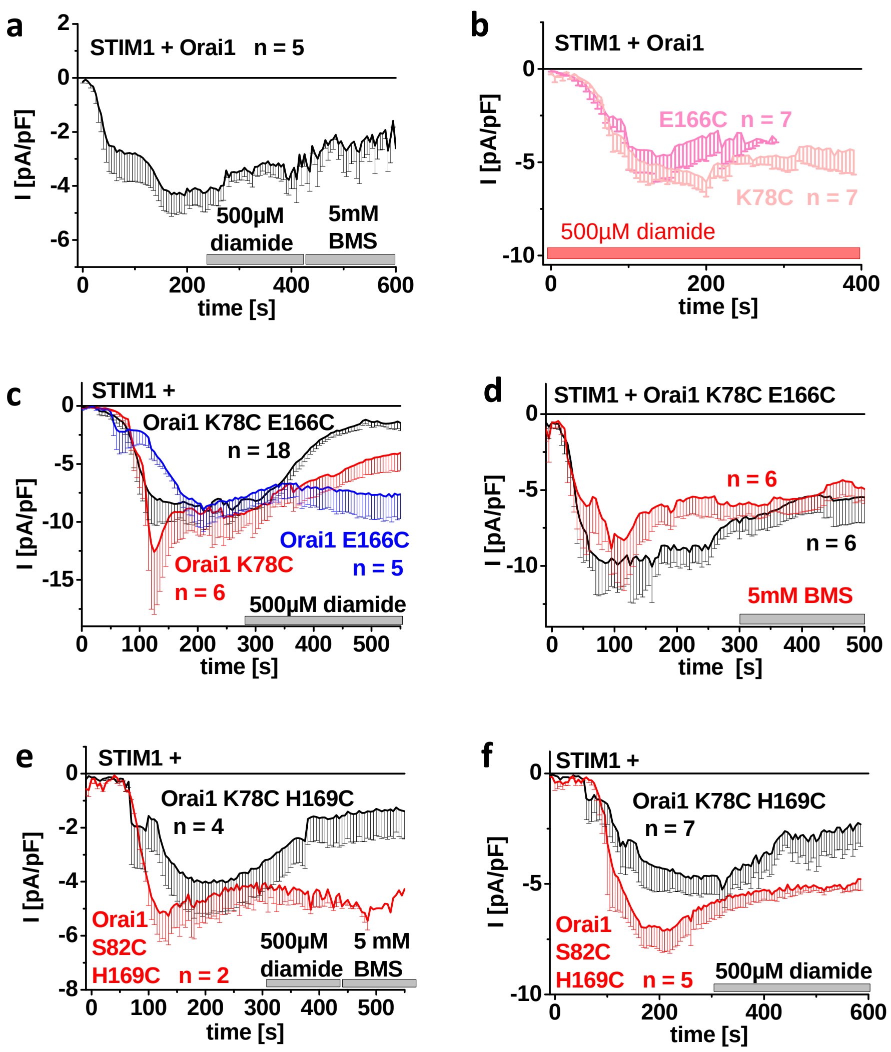

Supplement: Supporting Information [file 10.1074_M117.812693_jbc.M117.812693-1.pdf]
